# Predictive Value of Triglyceride-Glucose Index for In-hospital Mortality in Patients With Severe Fever With Thrombocytopenia Syndrome: A Multi-Center Observational Study

**DOI:** 10.3389/fmed.2021.768101

**Published:** 2022-01-04

**Authors:** Tingyu Zhang, Yuanni Liu, Ziruo Ge, Di Tian, Ling Lin, Zhenghua Zhao, Yi Shen, Xiaoli Yu, Yang Feng, Chunqian Qiang, Jianping Duan, Yanli Ma, Tianli Fan, Yongxiang Zhao, Zhihai Chen

**Affiliations:** ^1^Department of Infectious Disease, Beijing Ditan Hospital, Capital Medical University, Beijing, China; ^2^Department of Infectious Diseases, Yantai City Hospital for Infectious Disease, Yantai, China; ^3^Department of Infectious Diseases, Tai'an City Central Hospital, Tai'an, China; ^4^Department of Infectious Diseases, Dandong Infectious Disease Hospital, Dandong, China; ^5^Department of Infectious Diseases, Qing Dao No. 6 People's Hospital, Qingdao, China

**Keywords:** severe fever with thrombocytopenia syndrome, triglyceride-glucose index, in-hospital mortality, triglyceride, fasting blood glucose

## Abstract

**Background:** Triglyceride-glucose (TyG) index has been proposed as a reliable indicator for insulin resistance and proved to be closely associated with the severity and mortality risk of infectious diseases. It remains indistinct whether TyG index performs an important role in predicting in-hospital mortality in patients with severe fever with thrombocytopenia syndrome (SFTS).

**Methods:** The current study retrospectively recruited patients who were admitted for SFTS from January to December 2019 at five medical centers. TyG index was calculated in accordance with the description of previous study: Ln [fasting triglyceride (TG) (mg/dl) × fasting blood glucose (FBG) (mg/dl)/2]. The observational endpoint of the present study was defined as the in-hospital death.

**Results:** In total, 79 patients (64.9 ± 10.5 years, 39.2% female) who met the enrollment criteria were enrolled in the current study. During the hospitalization period, 17 (21.5%) patients died in the hospital. TyG index remained a significant and independent predictor for in-hospital death despite being fully adjusted for confounders, either being taken as a nominal [hazard ratio (*HR*) 5.923, 95% *CI* 1.208–29.036, *P* = 0.028] or continuous (*HR* 7.309, 95% *CI* 1.854–28.818, *P* = 0.004) variate. TyG index exhibited a moderate-to-high strength in predicting in-hospital death, with an area under the receiver operating characteristic curve (AUC) of 0.821 (95% *CI* 0.712–0.929, *P* < 0.001). The addition of TyG index displayed significant enhancement on the predictive value for in-hospital death beyond a baseline model, manifested as increased AUC (baseline model: 0.788, 95% *CI* 0.676–0.901 vs. + TyG index 0.866, 95% *CI* 0.783–0.950, *P* for comparison = 0.041), increased Harrell's C-index (baseline model: 0.762, 95% *CI* 0.645–0.880 vs. + TyG index 0.813, 95% *CI* 0.724–0.903, *P* for comparison = 0.035), significant continuous net reclassification improvement (NRI) (0.310, 95% *CI* 0.092–0.714, *P* = 0.013), and significant integrated discrimination improvement (0.111, 95% *CI* 0.008–0.254, *P* = 0.040).

**Conclusion:** Triglyceride-glucose index, a novel indicator simply calculated from fasting TG and FBG, is strongly and independently associated with the risk of in-hospital death in patients with SFTS.

## Background

Severe fever with thrombocytopenia syndrome (SFTS), caused by SFTS virus (SFTSV), has been recognized as a novel life-threatening infectious disease in recent years. SFTSV (New Bunyavirus) was isolated by Yu et al. and identified as a member of the genus phlebovirus in the Bunyaviridae family (1). SFTSV mainly infects humans through tick bites and may result in multiple organ dysfunction, particularly myocardial damage and kidney injury (2). Since it was first reported in China in 2009, the number of SFTS cases has increased rapidly, and related cases have been detected in neighboring countries, such as South Korea and Japan (1, 3, 4). Nowadays, SFTS has been involved in a wide range of regions throughout the world with a mortality rate of up to 30% (1), which makes it listed as an emerging infectious disease by the WHO in 2017 (5). For patients with severe situations, it may rapidly progress to multiple organ failure, then further lead to death within 1–2 weeks. Therefore, early risk stratification is of great clinical significance in identifying individuals with a high risk of poor outcomes and customizing specific strategies in line with the risk levels.

Triglyceride-glucose (TyG) index, which is simply calculated from fasting triglyceride (TG) and fasting blood glucose (FBG), has been proposed and considered as a reliable indicator for the estimation of insulin resistance levels (6). Certain previous studies have demonstrated that increased TyG index is significantly associated with an increased risk of chronic non-communicable diseases, consisting of diabetes mellitus, hypertension, non-alcoholic fatty liver disease, and cardiovascular disease (7–10). Furthermore, recent studies have indicated that TyG index plays important role in predicting the severity and mortality risk of infectious diseases, such as chronic hepatitis B and C (11, 12), and coronavirus disease-2019 (COVID-19) (13). It has been further revealed that the aggravation of acute or chronic infectious diseases caused by insulin resistance may be mainly mediated by multiple metabolic abnormalities and systemic inflammatory responses.

Currently, there is a relative lack of study targeting the association between TyG index and poor outcomes for patients with SFTS. The present study, therefore, was designed to investigate the value of TyG index in predicting the in-hospital mortality in patients who experienced SFTS, so as to provide useful information for the early identification of patients who were susceptible to adverse prognosis.

## Methods

### Study Population

**Figure 1:**
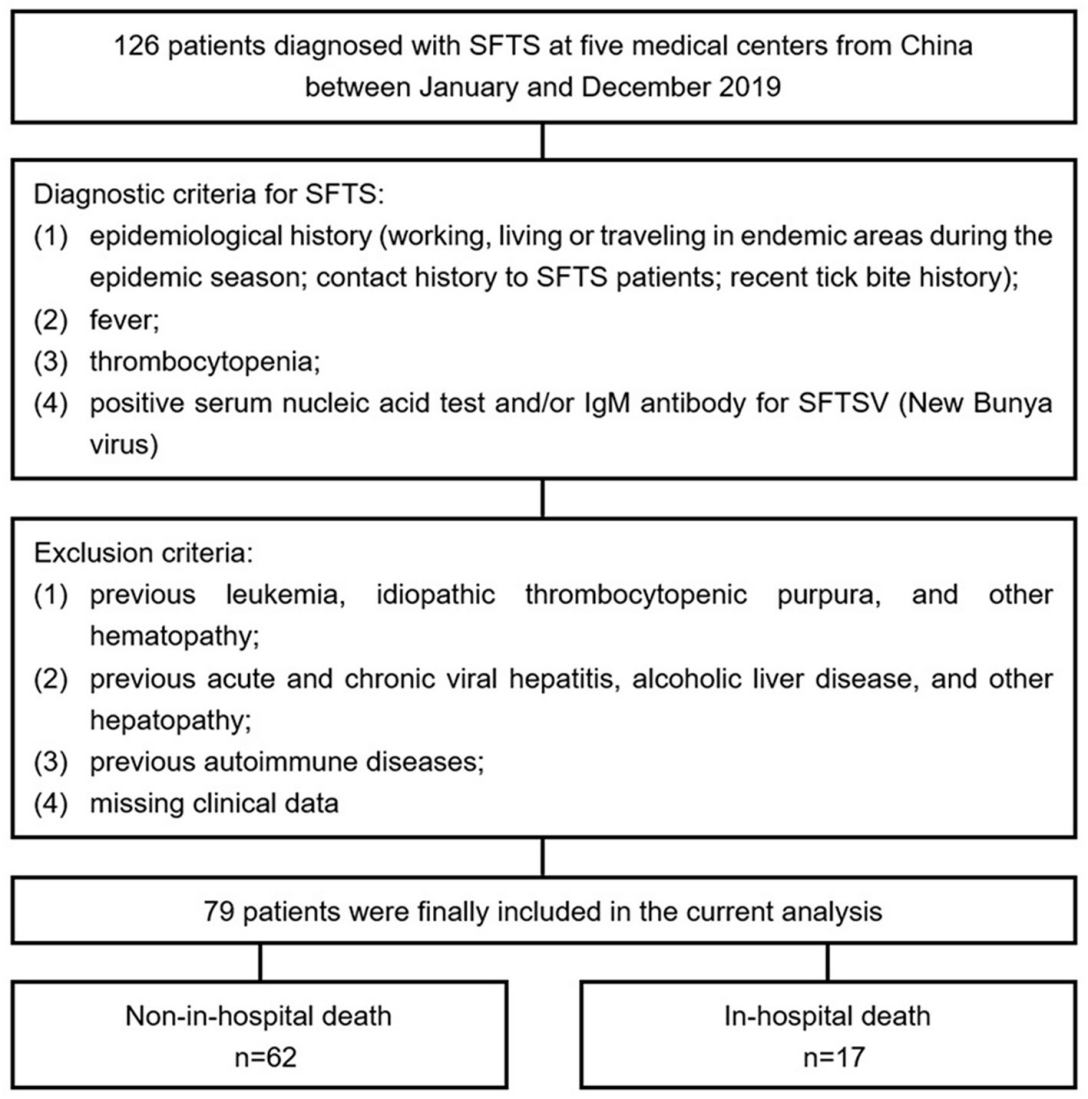
Flow diagram for the enrollment of study population. SFTS, severe fever with thrombocytopenia syndrome; SFTSV, SFTS virus.

A total of 126 patients diagnosed with SFTS at five medical centers from China (Beijing Ditan Hospital, Yantai City Hospital for Infectious Disease, Tai'an City Central Hospital, Qingdao No. 6 People's Hospital, and Dandong Infectious Disease Hospital) between January and December 2019 were retrospectively screened. The diagnostic criteria for SFTS were as follows: (1) epidemiological history (working, living, or traveling in endemic areas during the epidemic season; contact history to SFTS patients; and recent tick bite history); (2) fever; (3) thrombocytopenia; and (4) positive serum nucleic acid test and/or IgM antibody for SFTSV (New Bunyavirus). Exclusion criteria included were as follows: (1) previous leukemia, idiopathic thrombocytopenic purpura, and other hematopathies; (2) previous acute and chronic viral hepatitis, alcoholic liver disease, and other hepatopathies; (3) previous autoimmune diseases; and (4) missing clinical data. Ultimately, 79 patients who met the enrollment criteria were included in the current analysis (Figure 1).

The study protocol was endorsed by the Clinical Research Ethics Committee of Beijing Ditan Hospital, Capital Medical University. All subjects were informed and agreed to participate in the present study.

### Data Collection and Definitions

Demographic characteristics and clinical manifestations were acquired from a self-reported questionnaire and then entered into an established database by trained personnel who were blinded to the study protocol. The first laboratory examination during hospitalization, such as white blood cell count (WBC), neutrophil count (NEU), neutrophil percentage (NEU%), lymphocyte count (LYM), lymphocyte percentage (LYM%), platelet count (PLT), hemoglobin (Hb), T-lymphocyte (T-LYM), CD4 positive T-lymphocyte (CD4+ T-LYM), CD8 positive T-lymphocyte (CD8+ T-LYM), prothrombin time (PT), activated partial thromboplastin time (APTT), alanine aminotransferase (ALT), aspartate aminotransferase (AST), total bilirubin (TBIL), creatinine, blood urea nitrogen (BUN), creatine kinase (CK), lactic dehydrogenase (LDH), and C-reactive protein (CRP), were used for the current analysis. TG and FBG were examined by using peripheral venous blood samples extracted at ≥8 h fasting state. The formula for calculation of TyG index was conformed with the former study: Ln [fasting TG (mg/dl) × FBG (mg/dl)/2] (14).

The observational endpoint of the present study was defined as the in-hospital death. The time of death was defined as the period from symptom onset to in-hospital death occurred.

### Statistical Analysis

All data analyses were conducted with SPSS 26.0 (IBM, Armonk, NY, USA), MedCalc 19.1 (Ostend, Belgium), and R Programming Language 3.6.3. A two-sided *P* < 0.05 was regarded as statistical significance.

Continuous parameters with normal or abnormal distribution were presented as mean with SD or median with interquartile range (IQR), comparisons of which were performed by *T*-test or Mann–Whitney *U*-test respectively. Nominal parameters were presented as numbers with percentages and compared by chi-square test (with or without continuity correction) or Fisher's exact test correspondingly.

Kaplan–Meier analysis was performed to evaluate the cumulative survival rates of groups with lower and higher median of TyG index. The differences between groups were examined by using the log-rank test. Univariate Cox regression analyses were performed to identify the potential risk predictors of in-hospital death. Then, three multivariate models, variates of which were selected based on the results of univariate analysis and clinical experience, were established to investigate the independent role of TyG index in predicting in-hospital death. Variates with underlying collinearity were not introduced into models simultaneously. The hazard ratio (*HR*) and 95% *CI* for in-hospital death were evaluated by regarding the TyG index as a nominal or continuous variate, respectively.

The predictive performance of TyG index for in-hospital death was further evaluated by receiver operating characteristic (ROC) analysis whereby the area under the ROC curves (AUCs) and the optimal cut-off value were determined. At the level of optimal cut-off value, sensitivity, specificity, positive predictive value (PPV), and negative predictive value (NPV) for predicting the in-hospital death were calculated, respectively. Additionally, to evaluate the incremental effects of TyG index on risk prediction based on the model of established risk factors, the AUC and the Harrell's C-index for respective models were assessed and compared by *Z*-test. In addition, continuous net reclassification improvement (NRI) and integrated discrimination improvement (IDI) were also calculated to evaluate the incremental ability of TyG index on risk reclassification and discrimination.

## Results

In total, 79 patients with SFTS who met the inclusion and exclusion criteria were ultimately included in this study, with a mean age of 64.9 ± 10.5 years, including 48 men (60.8%) and 31 women (39.2%). During the hospitalization period, 17 patients died in hospital, with a mortality rate of 21.5%.

### Baseline Characteristics of the Study Population

Patients were divided into two groups based on the occurrence of in-hospital death. As manifested in Table 1, there were no significant discrepancies in demographic and medical characteristics between the non-in-hospital death and the in-hospital death group. Similarly, as for clinical manifestations, no significant differences emerged between the two groups (Table 2). In terms of laboratory examinations, patients who died in hospital showed higher levels of ALT, AST, creatine, BUN, FBG, CK, LDH, and TG, while lower levels of PLT, T-LYM, CD4+ T-LYM, and CD8+ T-LYM. The differences in WBC, NEU, NEU%, LYM, LYM%, Hb, PT, APTT, TBIL, and CRP were not significant (Table 3).

**Table 1:** Baseline demographic and medical characteristics of the study population.

	**Total population** ***n* = 79**	**Non-in-hospital death *n* = 62**	**In-hospital death** ***n* = 17**	***P***
Age, years	64.9 ± 10.5	63.9 ± 10.6	68.6 ± 9.9	0.106
Gender, female (%)	31 (39.2)	25 (40.3)	6 (35.3)	0.707
Residence (%)				0.591
Rural	69 (87.3)	53 (85.5)	16 (94.1)	
Urban	10 (12.7)	9 (14.5)	1 (5.9)	
History of bites (%)	20 (25.3)	16 (25.8)	4 (23.5)	>0.999
**Medical history (%)**				
Hypertension	17 (21.5)	13 (21.0)	4 (23.5)	>0.999
Diabetes mellitus	5 (6.3)	4 (6.5)	1 (5.9)	>0.999
Cardiovascular disease	6 (7.6)	5 (8.1)	1 (5.9)	>0.999
Cerebrovascular disease	5 (6.3)	4 (6.5)	1 (5.9)	>0.999
Time from onset to admission, days	6.8 ± 3.8	6.8 ± 4.0	6.5 ± 3.4	0.771

**Table 2:** Baseline clinical manifestations of the study population.

	**Total population** ***n* = 79**	**Non-in-hospital death *n* = 62**	**In-hospital death** ***n* = 17**	***P***
**General manifestations (%)**				
Pyrexia	78 (98.7)	61 (98.4)	17 (100.0)	>0.999
Shiver	30 (38.0)	25 (40.3)	5 (29.4)	0.412
Fatigue	60 (75.9)	44 (71.0)	16 (94.1)	0.097
Sore muscle	21 (26.6)	18 (29.0)	3 (17.6)	0.528
Arthralgia	8 (10.1)	6 (9.7)	2 (11.8)	>0.999
Lymphadenectasis	8 (10.1)	4 (6.5)	4 (23.5)	0.107
Petechiae	3 (3.8)	2 (3.2)	1 (5.9)	>0.999
**Respiratory manifestations (%)**				
Cough	12 (15.2)	7 (11.3)	5 (29.4)	0.065
Expectoration	5 (6.3)	2 (3.2)	3 (17.6)	0.109
Dyspnea	6 (7.6)	5 (8.1)	1 (5.9)	>0.999
**Gastrointestinal manifestations (%)**				
Anorexia	45 (57.0)	36 (58.1)	9 (52.9)	0.705
Nausea	32 (40.5)	23 (37.1)	9 (52.9)	0.238
Emesis	19 (24.1)	12 (19.4)	7 (41.2)	0.062
Abdominal pain	2 (2.5)	2 (3.2)	0 (0.0)	>0.999
Diarrhea	16 (20.3)	15 (24.2)	1 (5.9)	0.186
**Neurological manifestations (%)**				
Dizzy	17 (21.5)	12 (19.4)	5 (29.4)	0.371
Headache	14 (17.7)	11 (17.7)	3 (17.6)	>0.999
Disturbance of consciousness	5 (6.3)	3 (4.8)	2 (11.8)	0.634

**Table 3:** Baseline laboratory results of the study population.

	**Total population** ***n* = 79**	**Non-in-hospital death** ***n* = 62**	**In-hospital death** ***n* = 17**	***P***
WBC, ×10^9^/L	3.2 (1.8, 5.5)	3.3 (1.9, 5.6)	3.0 (1.3, 5.3)	0.381
NEU, ×10^9^/L	1.9 (1.1, 3.0)	1.9 (1.2, 3.5)	1.8 (0.7, 2.7)	0.390
NEU%, %	63.8 (50.9, 75.6)	63.7 (50.9, 77.6)	63.8 (51.8, 69.0)	0.512
LYM, ×10^9^/L	0.9 (0.5, 1.6)	0.9 (0.5, 1.6)	0.9 (0.5, 1.7)	0.967
LYM%, %	26.8 (19.4, 39.5)	26.5 (18.2, 39.7)	31.1 (24.2, 39.1)	0.355
PLT, ×10^9^/L	55.0 (34.0, 72.0)	58.5 (40.8, 73.0)	38.0 (27.5, 54.0)	0.008
Hb, g/L	137.0 (127.0, 148.0)	137.5 (124.8, 146.3)	137.0 (128.5, 156.0)	0.421
T-LYM, cells/uL	746.0 (386.0, 1036.0)	806.5 (482.3, 1221.3)	405.0 (279.5, 799.0)	0.005
CD4+ T-LYM, cells/uL	318.0 (185.0, 460.0)	372.5 (208.0, 488.0)	187.0 (144.0, 304.5)	0.003
CD8+ T-LYM, cells/uL	268.0 (146.0, 465.0)	342.0 (183.0, 494.8)	172.0 (102.5, 276.5)	0.005
PT, s	11.9 ± 1.3	11.8 ± 1.1	12.2 ± 1.7	0.248
APTT, s	43.1 (38.7, 52.7)	44.0 (38.7, 52.3)	42.2 (39.0, 54.6)	0.957
ALT, U/L	88.0 (58.0, 138.0)	82.5 (50.8, 119.8)	135.0 (71.0, 188.5)	0.035
AST, U/L	169.0 (122.0, 294.0)	161.0 (97.8, 258.8)	239.0 (154.5, 392.5)	0.049
TBIL, mg/dL	10.0 (7.2, 15.9)	10.6 (8.0, 14.9)	7.7 (6.3, 19.9)	0.228
Creatine, umol/L	68.0 (57.0, 83.6)	66.4 (55.8, 80.7)	79.6 (69.2, 127.7)	0.012
BUN, mmol/L	5.6 (3.6, 7.6)	5.0 (3.3, 7.3)	7.3 (5.5, 9.0)	0.033
FBG, mmol/L	6.4 (5.6, 7.8)	6.1 (5.5, 6.8)	8.6 (6.8, 9.8)	<0.001
CK, U/L	434.0 (170.0, 1280.0)	289.7 (143.3, 912.8)	1504.0 (564.5, 2975.2)	<0.001
LDH, U/L	819.0 (466.2, 1210.0)	695.0 (446.9, 1162.4)	994.0 (857.5, 1382.6)	0.003
CRP, mg/L	3.7 (1.8, 7.3)	3.1 (1.8, 6.9)	5.7 (2.4, 11.7)	0.185
TG, mmol/L	1.9 (1.3, 2.6)	1.7 (1.1, 2.2)	2.6 (1.9, 3.5)	0.001

*WBC, white blood cell; NEU, neutrophil; NEU%, neutrophil percentage; LYM, lymphocyte; LYM%, lymphocyte percentage; PLT, platelet; Hb, hemoglobin; PT, prothrombin time; APTT, activated partial thromboplastin time; ALT, alanine aminotransferase; AST, aspartate aminotransferase; TBIL, total bilirubin; BUN, blood urea nitrogen; FBG, fasting blood glucose; CK, creatinine kinase; LDH, lactate dehydrogenase; CRP, C-reactive protein; TG, triglyceride*.

### Association Between TyG Index and In-hospital Death

**Figure 2:**
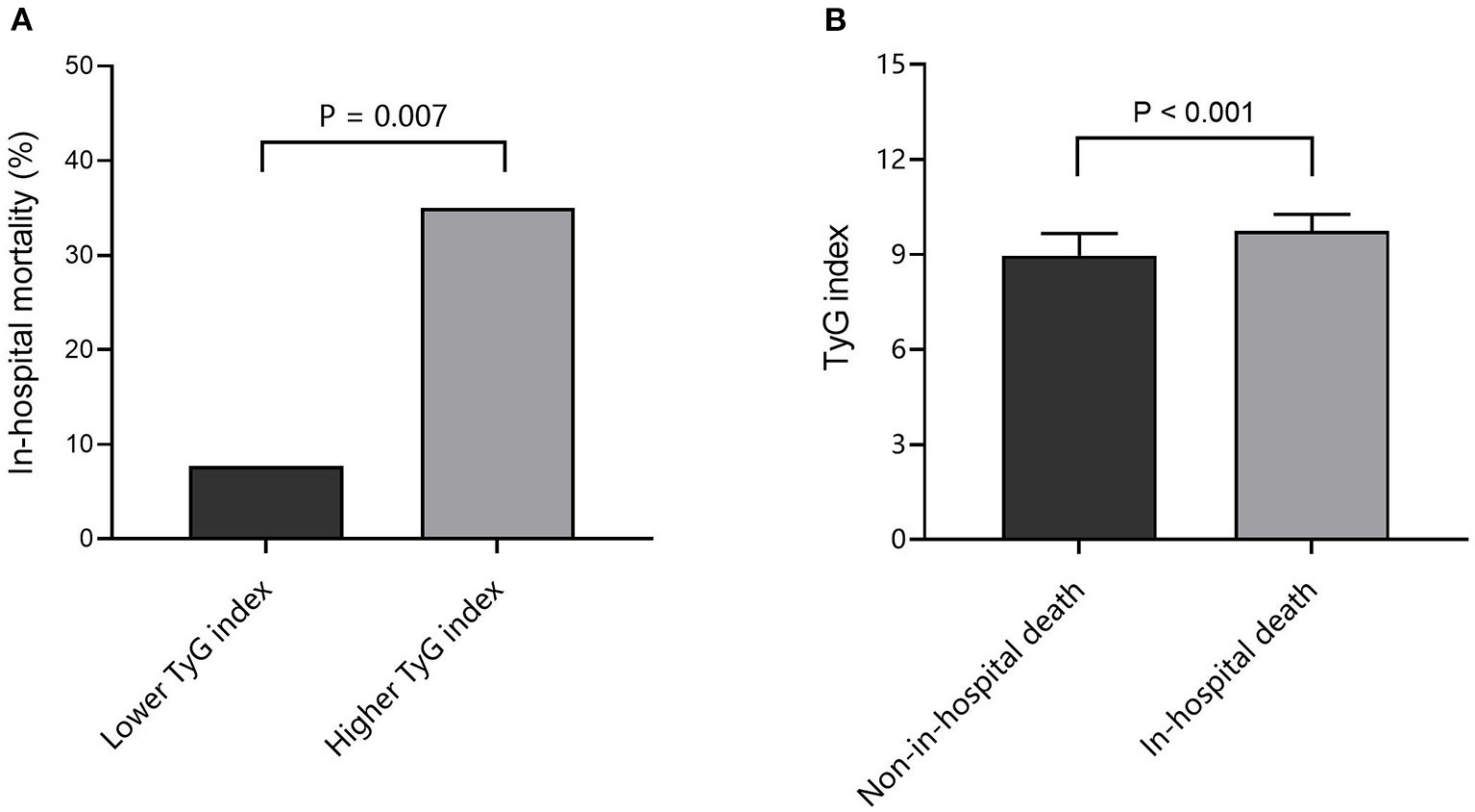
Association between TyG index and in-hospital death. **(A)** Comparison of in-hospital mortality at different TyG index levels; **(B)** Comparison of TyG index between non-in-hospital death and in-hospital death cases. TyG, triglyceride-glucose.

As shown in Figure 2A, the in-hospital mortality of SFTS patients with higher median of TyG index (median: 9.05) was significantly higher than that of patients with lower median [14 (35.0%) vs. 3 (7.7%), *P* = 0.007]. On the other hand, the level of TyG index in SFTS patients who died in hospital was significantly higher than that in patients who survived (9.74 ± 0.53 vs. 8.96 ± 0.70, *P* < 0.001) (Figure 2B).

### TyG Index as an Independent Predictor of In-hospital Death

**Figure 3:**
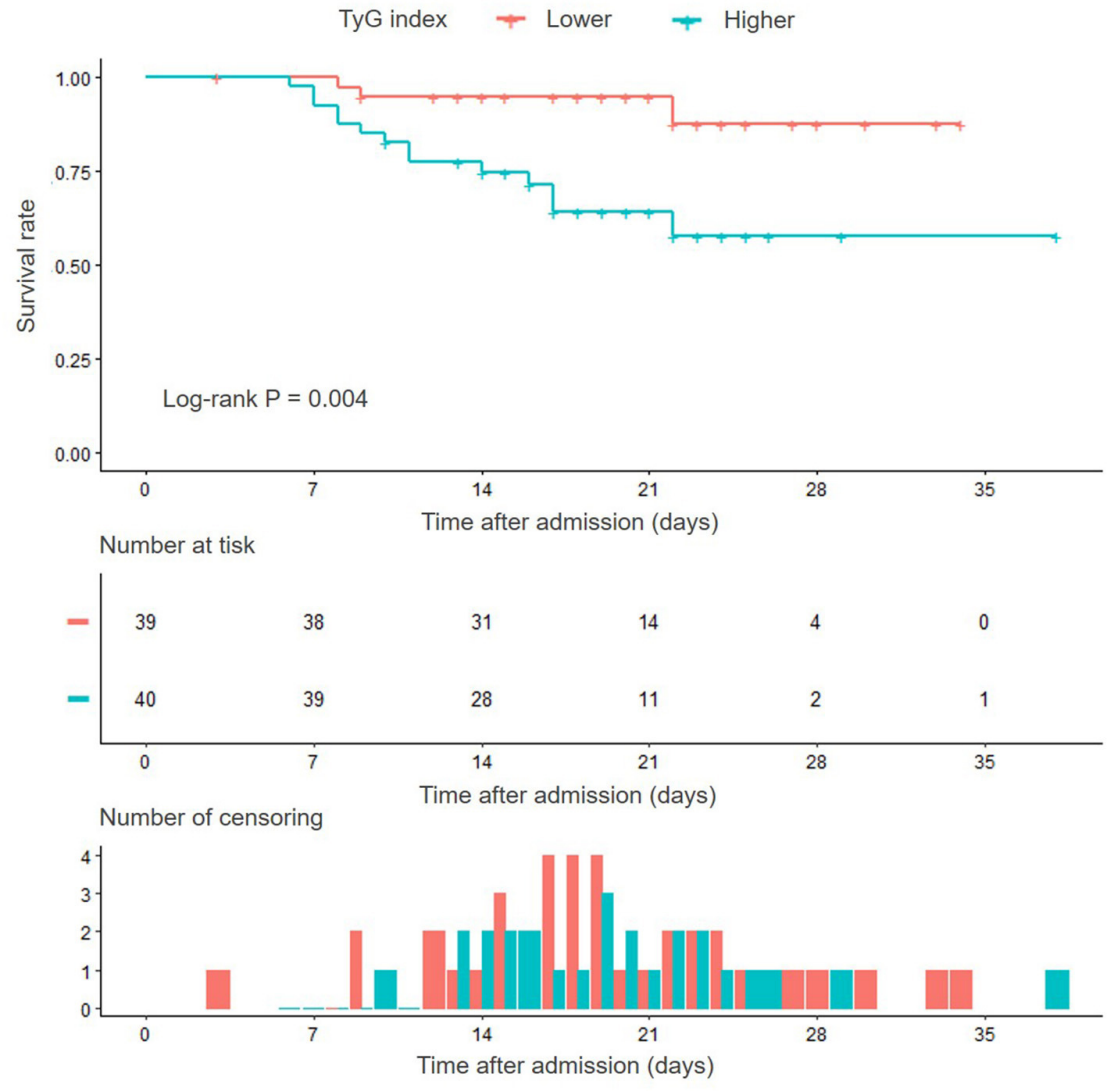
Kaplan–Meier survival curves according to the median of TyG index. TyG, triglyceride-glucose.

Kaplan–Meier survival analysis showed that the cumulative in-hospital survival rate of patients with SFTS with higher TyG index was significantly lower than that of patients with lower TyG index (Log-rank *P* = 0.004) (Figure 3). Moreover, univariate Cox regression analysis confirmed the significant predictive value of TyG index for in-hospital death, despite regarding TyG index as a nominal variable (unadjusted *HR* for taking lower median as reference 5.148, 95% *CI* 1.478–17.936, *P* = 0.010) or continuous variable (unadjusted *HR* for per 1-unit increase 4.572, 95% *CI* 2.064–10.127, *P* < 0.001).

The results from univariate Cox regression analysis revealed that PLT, T-LYM, CD4+ T-LYM, CD8+ T-LYM, AST, FBG, CK, LDH, and TG were potential risk predictors of in-hospital death (Supplementary Table 1). Considering the results of univariate analysis and clinical experience, three multivariate models were established to investigate whether TyG index is an independent predictor of in-hospital death. With the adjustment of confounding factors, increased TyG index was independently and robustly associated with an increased risk of in-hospital death, either evaluating *HR* by taking the lower median of TyG index as reference (Model 1: *HR* 5.427, 95% *CI* 1.548–19.022, *P* = 0.008; Model 2: *HR* 6.288, 95% *CI* 1.746–22.651, *P* = 0.005; Model 3: *HR* 5.923, 95% *CI* 1.208–29.036, *P* = 0.028) or by examining 1-unit increase of TyG index (Model 1: *HR* 4.367, 95% *CI* 1.961–9.726, *P* < 0.001; Model 2: *HR* 6.782, 95% *CI* 2.336–19.686, *P* < 0.001; Model 3: *HR* 7.309, 95% *CI* 1.854–28.818, *P* = 0.004) (Table 4).

**Table 4:** The predictive value of triglyceride-glucose (TyG) index for in-hospital death.

	**TyG index as nominal**			**TyG index as continuous**		
	**variate^a^**			**variate^b^**		
	**HR**	**95% CI**	***P***	**HR**	**95% CI**	***P***
Crude model	5.148	1.478–17.936	0.010	4.572	2.064–10.127	<0.001
Model 1	5.427	1.548–19.022	0.008	4.367	1.961–9.726	<0.001
Model 2	6.288	1.746–22.651	0.005	6.782	2.336–19.686	<0.001
Model 3	5.923	1.208–29.036	0.028	7.309	1.854–28.818	0.004

*Model 1: adjusted for age and gender.*

*Model 2: adjusted for Model 1 and hypertension, diabetes mellitus, cardiovascular disease, cerebrovascular disease, and neurological manifestations.*

*Model 3: adjusted for Model 2 and NEU%, PLT, CD4+ T-LYM, AST, BUN, CK, and LDH.*

*TyG, triglyceride-glucose; HR, hazard ratio.*

a *HR was evaluated by taking the lower median of TyG index as reference.*

b *HR was evaluated by examining 1-unit increase of TyG index*.

### Predictive Performance of TyG Index for In-hospital Death

**Figure 4:**
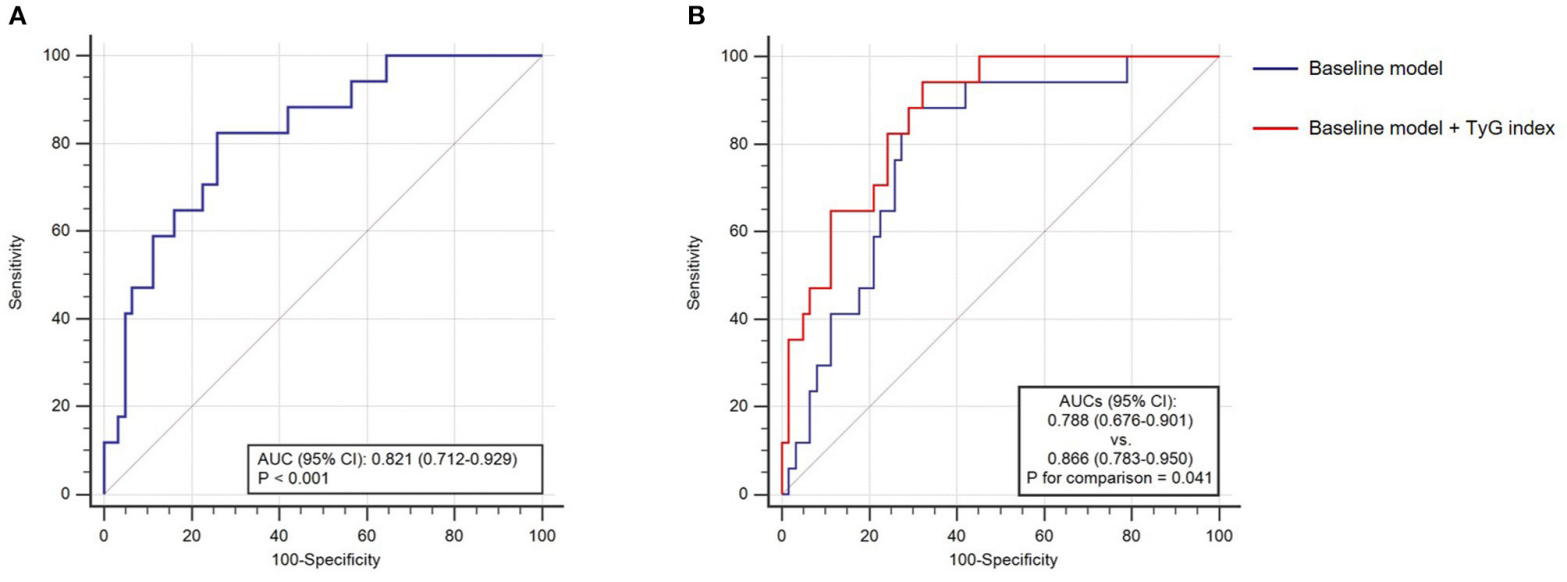
The receiver operating characteristics (ROC) curve analysis. **(A)** The predictive value of TyG index for in-hospital death; **(B)** the incremental effect of TyG index on the prediction of in-hospital death. AUC, area under the ROC curve; *CI*, confidence interval; and TyG, triglyceride-glucose.

The predictive performance of TyG index for in-hospital death was further evaluated by ROC curve analysis. As shown in Figure 4A, TyG index displayed a moderate-to-high strength in predicting in-hospital death, with an AUC of 0.821 (95% *CI* 0.712–0.929, *P* < 0.001). The optimal cut-off value was 9.31, under which the sensitivity (95% *CI*), specificity (95% *CI*), PPV (95% *CI*), and NPV (95% *CI*) for predicting the in-hospital death were 82.4% (56.6–96.2%), 74.2% (61.5–84.5%), 46.7% (35.2–58.5%), and 93.9% (84.5–97.7%), respectively.

### Incremental Effect of TyG Index on the Prediction of In-hospital Death

After introducing TyG index into a baseline model, such as risk factors identified by former authoritative study (age, neurological manifestations, LDH, AST, BUN, and NEU%) (15), there was a significant incremental effect on the predictive performance for in-hospital death, manifested as an increased AUC (baseline model: 0.788, 95% *CI* 0.676–0.901 vs. baseline model + TyG index 0.866, 95% *CI* 0.783–0.950, *P* for comparison = 0.041) (Figure 4B). Moreover, the addition of TyG index to the baseline model contributed to an increased Harrell's C-index (baseline model: 0.762, 95% *CI* 0.645–0.880 vs. baseline model + TyG index 0.813, 95% *CI* 0.724–0.903, *P* for comparison = 0.035), significant continuous NRI (0.310, 95% *CI* 0.092–0.714, *P* = 0.013), and significant IDI (0.111, 95% *CI* 0.008–0.254, *P* = 0.040) (Table 5).

**Table 5:** Incremental effect of TyG index on the prediction of in-hospital death.

	**Harrell's C-index**			**Continuous NRI**			**IDI**		
	**Est**.	**95% CI**	***P***	**Est**.	**95% CI**	***P***	**Est**.	**95% CI**	***P***
Baseline model^a^	0.762	0.645–0.880	-	-	-	-	-	-	-
+ TyG index	0.813	0.724–0.903	0.035	0.310	0.092–0.714	0.013	0.111	0.008–0.254	0.040

*TyG, triglyceride-glucose; NRI, net reclassification improvement; and IDI, integrated discrimination improvement.*

a *The baseline model including age, neurological manifestations, LDH, AST, BUN, and NEU%*.

## Discussion

As we know, this is the first study to investigate the predictive value of TyG index for in-hospital death in patients with SFTS. The main findings are summarized as follows. (1) Patients with higher TyG index exhibited higher mortality rate, and in reverse, the TyG index in patients with in-hospital death was significantly higher than that of patients without; (2) Despite the adjustment for confounding risk factors, TyG index remained a significant and independent predictor for in-hospital death, either being taken as a nominal or continuous variate; (3) TyG index displayed a moderate-to-high strength in predicting in-hospital death; (4) After being introduced into a baseline model, TyG index showed an incremental effect on the prediction of in-hospital death.

In China, SFTS has been reported mainly in rural regions in the Eastern and Central provinces. SFTSV, which causes SFTS, is a novel phlebovirus in the Bunyaviridae family. The genome of SFTSV consists of three minus-strand RNA segments: large, medium, and small segments (16). SFTSV can invade multiple organs and propagate in the cytoplasm of host cells, such as the spleen, liver, and kidney (17). For critically ill patients with SFTS patients, their clinical symptoms usually exacerbate during the acute phase of the disease and quickly proceed to multiple organ dysfunction syndromes, disseminated intravascular coagulation, and ending in death ~1–2 weeks after the onset of the disease (18). No vaccines or effective drugs against SFTSV have yet been developed. Therefore, early identification of high-risk patients has important clinical significance for improving the prognosis of SFTS. In the early stages of SFTS, patients with severe conditions may have significantly increased markers compared with patients with mild conditions. Our study found that the TyG index in patients with in-hospital death was significantly higher than that of patients without, indicating that the TyG index may be an effective indicator for early identification of patients who are prone to poor outcomes.

As a novel surrogate marker of insulin resistance, TyG index has been extensively used to predict the risk and severity of several diseases. It has been demonstrated that TyG index is a significant predictor for the risk of chronic non-communicable diseases, such as type 2 diabetes mellitus (T2DM), hypertension, cardiovascular disease, and non-alcoholic fatty liver disease (7–10). As for infectious diseases, TyG index has been revealed to be closely associated with the severity of chronic hepatitis B and C, and directly correlated to the viral load of chronic hepatitis C (11, 12). A recent study from China, which investigated the association between TyG index and COVID-19, showed that an increased level of TyG index is closely related to a higher incidence of severe COVID-19. In addition, TyG index could predict the mortality risk of patients with COVID-19 (13). These studies suggest the great potential of TyG index to be served as a novel risk predictor for patients with infectious diseases. The present study, which first elucidated the predictive value of TyG index for in-hospital death in patients with SFTS, fills the gap of previous research in this field. The findings of this study provide a simple and accessible pathway for early identification of patients who are susceptible to develop adverse prognosis, which is of great clinical significance for customizing individualized intervention strategies that match the risk levels. Furthermore, a significant incremental effect on the prediction of in-hospital death emerged after introducing TyG index into a baseline model, such as risk factors identified by former authoritative study, indicating that TyG index may contribute more information to prognostic prediction on the basis of established risk factors.

Although the present study has revealed the close relationship between TyG index and in-hospital outcomes of patients with SFTS, the potential mechanisms involved in this association remained indistinct. Since it has been extensively demonstrated that TyG index is a significant surrogate marker of insulin resistance, we hypothesized that the reason for the association may be attributed to insulin resistance. Insulin resistance plays a vital role in the development of dyslipidemia and hyperglycemia, which makes them the two most important hallmarks of insulin resistance. The formula of TyG index is composed of fasting FBG and TG, while recent evidence has proved that FBG and TG play important role in the progress of SFTS.

Previous studies have shown that patients with SFTS with diabetes and/or increased FBG exhibit higher mortality (19, 20), indicating the important role of glucose metabolism in disease progression. Study has revealed that the plasma levels of glucagon, adrenaline, and glucocorticoid in patients with severe infections are significantly increased, which can further lead to disturbance of glucose metabolism characterized as significant decrease in insulin sensitivity and increase in blood glucose (21). The increased level of blood glucose resulting from severe infections can activate inflammatory responses that cause deterioration of the disease. It has been demonstrated that the increased level of blood glucose promotes a sharp increase in the concentrations of inflammatory cytokines, such as interleukin-6, tumor necrosis factor α, and interleukin-18, and this effect was more pronounced in subjects with abnormal glucose tolerance (22). Moreover, study has proved that the application of antioxidant glutathione can prevent the short-term rise of inflammatory cytokines induced by hyperglycemia, suggesting that hyperglycemia might affect the concentration of cytokines through the oxidative mechanism. On the other hand, studies have confirmed that lipid metabolism plays a pivotal role in viral lifecycle, such as replication, membrane homeostasis, endocytosis, and exocytosis (23, 24). A recent study has proved that the synthesis pathway of cholesterol, fatty acids, and TG regulated by site-1 protease plays an important role in SFTSV replication and reproduction (25), indicating that the excessive synthesis of lipid may be responsible for the increase in SFTSV replication. *In vitro* experiment confirmed that the replication of SFTSV in cells is significantly decreased after the treatment with lovastatin and fenofibrate, which inhibits the synthesis of cholesterol and TG, while distinctly increased after being introduced into an environment with a high level of TG, which may be an important explanation for the association of TG with adverse prognosis in patients with SFTS (25).

Former studies have revealed that T2DM may impair the immune system by suppressing complement components, dysregulating innate immunocytes (dendritic cell, macrophage, neutrophil, natural killer cell, etc.), and inhibiting adaptive immunocytes (B lymphocyte, T lymphocyte, etc.), which makes individuals more susceptible to the infection by microbes or to severe conditions after infection (26). As the most significant pathogenesis of T2DM, it can be speculated that insulin resistance plays an important role in these processes. On the other hand, infection with a variety of viruses, such as human immunodeficiency virus (27), hepatitis C virus (28), murine gamma herpesvirus 68 (29), may inversely cause or exacerbate insulin resistance, thus leading to a vicious cycle. Additionally, it has been demonstrated that insulin resistance is significantly associated with systemic inflammatory responses (30, 31), which determine the severity and outcomes of infectious diseases. In general, since the current study is hypothesis-generating, further research is required to explore the underlying pathophysiological process and mechanism mediating the association between TyG index and adverse prognosis of patients with SFTS.

Some limitations need to be acknowledged. First, as a retrospective, observational study with relatively small sample size, the statistical strength may be limited. Second, although the measurement of viral load and/or antibody titer can provide useful information, it was not available in the database. Third, the observational endpoint of the present study was defined as the in-hospital death, adverse events obtained from follow-up after discharge may offer more valuable information. Finally, situations, such as stress and fasting duration may have non-negligible effects on the estimation of TyG index.

## Conclusion

Triglyceride-glucose index is strongly and independently associated with the risk of in-hospital death in patients with SFTS. TyG index, which is derived from the fasting TG and FBG, may provide valuable information on the basis of established risk factors in identifying patients at high risk of developing in-hospital adverse prognosis, thus can be served as an accessible and useful risk predictor in clinical practice. The findings require further large-scale and prospective studies to confirm.

## Data Availability Statement

The raw data supporting the conclusions of this article will be made available by the authors, without undue reservation.

## Ethics Statement

The studies involving human participants were reviewed and approved by Clinical Research Ethics Committee of Beijing Ditan Hospital, Capital Medical University. The patients/participants provided their written informed consent to participate in this study.

## Author Contributions

TZ and ZC made substantial contributions to study design, data analysis, and manuscript writing. ZG and DT made substantial contributions to study design, intellectual direction, and manuscript revision. YL made substantial contributions to data collection, data follow-up, and manuscript revision. DT, LL, ZZ, YS, XY, YF, CQ, JD, YM, TF, and YZ made substantial contributions to data collection and follow-up. All authors have read and approved the final manuscript.

## Funding

This study was supported by the National Natural Science Foundation of China (82072295).

## Conflict of Interest

The authors declare that the research was conducted in the absence of any commercial or financial relationships that could be construed as a potential conflict of interest.

## Publisher's Note

All claims expressed in this article are solely those of the authors and do not necessarily represent those of their affiliated organizations, or those of the publisher, the editors and the reviewers. Any product that may be evaluated in this article, or claim that may be made by its manufacturer, is not guaranteed or endorsed by the publisher.

## Abbreviations

SFTS: severe fever with thrombocytopenia syndrome
SFTSV: SFTS virus
TyG: Triglyceride-glucose
TG: triglyceride
FBG: fasting blood glucose
COVID-19: coronavirus disease-2019
WBC: white blood cell count
NEU: neutrophil count
NEU%: neutrophil percentage
LYM: lymphocyte count
LYM%: lymphocyte percentage
PLT platelet count
Hb: hemoglobin
T-LYM: T-lymphocyte
CD4+ T-LYM: CD4 positive T-lymphocyte
CD8+ T-LYM: CD8 positive T-lymphocyte
PT: prothrombin time
APTT: activated partial thromboplastin time
ALT: alanine aminotransferase
AST: aspartate aminotransferase
TBIL: total bilirubin
BUN: blood urea nitrogen
CK: creatine kinase
LDH: lactic dehydrogenase
CRP: C-reactive protein
HR: hazard ratio
CI: 95% confidence interval
ROC: receiver operating characteristic
AUC: area under the ROC curve
PPV: positive predictive value
NPV: negative predictive value
NRI: net reclassification improvement
IDI: integrated discrimination improvement
T2DM: type 2 diabetes mellitus.
