# Supplementary material for: Predictive Value of Triglyceride-Glucose Index for In-hospital Mortality in Patients With Severe Fever With Thrombocytopenia Syndrome: A Multi-Center Observational Study
Source: Front Med (Lausanne). 2022 Jan 4;8:768101. doi: 10.3389/fmed.2021.768101 (PMC8763701; doi:10.3389/fmed.2021.768101)
Supplement: Supplementary file 1 [file Table_1.DOCX]

## Table S1. Univariate COX proportional hazard regression evaluating potential predictors of in-hospital death

|  | HR | 95% CI | P |
| --- | --- | --- | --- |
| Age | 1.041 | 0.992-1.094 | 0.104 |
| Gender | 0.842 | 0.311-2.278 | 0.734 |
| Residence | 2.292 | 0.303-17.318 | 0.421 |
| History of bites | 0.928 | 0.303-2.847 | 0.897 |
| Hypertension | 1.024 | 0.333-3.147 | 0.967 |
| Diabetes mellitus | 1.307 | 0.170-10.026 | 0.797 |
| Cardiovascular disease | 0.907 | 0.119-6.917 | 0.925 |
| Cerebrovascular Disease | 0.845 | 0.112-6.380 | 0.870 |
| Time from onset to admission | 0.952 | 0.834-1.087 | 0.467 |
| Neurological manifestations | 2.106 | 0.812-5.465 | 0.126 |
| WBC | 0.887 | 0.719-1.096 | 0.267 |
| NEU | 0.799 | 0.573-1.112 | 0.183 |
| NEU% | 0.995 | 0.967-1.024 | 0.748 |
| LYM | 0.875 | 0.444-1.724 | 0.700 |
| LYM% | 1.007 | 0.975-1.040 | 0.665 |
| PLT | 0.973 | 0.951-0.995 | 0.017 |
| Hb | 1.008 | 0.978-1.039 | 0.595 |
| T-LYM | 0.998 | 0.997-1.000 | 0.027 |
| CD4+ T-LYM | 0.995 | 0.992-0.999 | 0.010 |
| CD8+ T-LYM | 0.997 | 0.993-1.000 | 0.030 |
| PT | 1.333 | 0.891-1.994 | 0.162 |
| APTT | 1.018 | 0.988-1.049 | 0.232 |
| ALT | 1.003 | 0.999-1.006 | 0.103 |
| AST | 1.003 | 1.000-1.006 | 0.024 |
| TBIL | 1.033 | 0.983-1.085 | 0.196 |
| Creatine | 1.003 | 0.999-1.007 | 0.198 |
| BUN | 1.040 | 0.973-1.110 | 0.249 |
| FBG | 1.528 | 1.232-1.895 | <0.001 |
| CK | 1.000 | 1.000-1.000 | 0.011 |
| LDH | 1.001 | 1.000-1.001 | 0.028 |
| CRP | 1.025 | 0.977-1.076 | 0.310 |
| TG | 1.775 | 1.233-2.556 | 0.002 |

WBC white blood cell, NEU neutrophil, NEU% neutrophil percentage, LYM lymphocyte, LYM% lymphocyte percentage, PLT platelet, Hb hemoglobin, PT prothrombin time, APTT activated partial thromboplastin time, ALT alanine aminotransferase, AST aspartate aminotransferase, TBIL total bilirubin, BUN blood urea nitrogen, FBG fasting blood glucose, CK creatinine kinase, LDH lactate dehydrogenase, CRP C-reactive protein, TG triglyceride, HR hazard ratio, CI confidence interval
